# Endoscopic transposition with the hernial sac (eTHS) in ventral hernia repair - technical description

**DOI:** 10.1590/0100-6991e-20202672

**Published:** 2021-02-26

**Authors:** LEONARDO EMILIO DA-SILVA, RENATO MIRANDA DE MELO

**Affiliations:** 1 - Universidade Federal de Goiás, Professor Associado, Faculdade de Medicina - Departamento de Cirurgia - Goiânia - GO - Brasil; 2 - Colégio Brasileiro de Cirurgiões, Titular - Goiânia - GO - Brasil

**Keywords:** Laparoscopy, Hernia, Ventral, Bioprosthesis, Peritoneum, Herniorrhaphy, Laparoscopia, Hérnia Ventral, Bioprótese, Peritônio, Herniorrafia

## Abstract

Incisional hernia is a late complication of the most frequent after abdominal surgeries, with resulting morbidity that can worsen the condition. The treatment has been done both by open techniques, using screens or not, and by laparoscopic and robotic methods, which use them systematically. However, introducing a permanent foreign body into the tissues requires more surgical time, despite not closing the parietal defect in most cases and a higher risk of infections. New technologies have been trying to improve these results, with absorbable prostheses (biological or synthetic), but their high cost and recurrences remain a severe problem. Even so, standard repair establishes reinforcement with screens, routine, and whether the approach is traditional or mini-invasive. The authors report their first case of endoscopic repair of incisional hernia, which occurred two years ago, with a Brazilian technique already fifty years old: the transposition with the hernia sac proposed by Prof. Alcino Lázaro da Silva in 1971.

## RESUMO

A hérnia incisional é uma das complicações tardias mais frequentes, após operações abdominais, resultando em alta morbidade. O tratamento tem sido feito tanto por técnicas abertas, com telas ou sem, quanto por métodos laparoscópicos e robóticos, que utilizam próteses de forma sistemática. No entanto, a introdução de um corpo estranho permanente entre os tecidos requer mais tempo cirúrgico, não fecha o defeito parietal na maioria dos casos e está associado a maior risco de infecções. Novas tecnologias têm sido utilizadas para melhorar esses resultados, empregando próteses absorvíveis (biológicas ou sintéticas), mas o alto custo e as recidivas continuam sendo um grave problema. No entanto, a reparação padrão rotineiramente estabelece reforço com telas, seja na abordagem tradicional ou minimamente invasiva. Os autores relatam o primeiro caso do reparo endoscópico de hérnia incisional, usando técnica brasileira com 50 anos de idade: a transposição com o saco herniário, conforme proposta pelo Prof. Alcino Lázaro da Silva, em 1971.

## INTRODUCTION

New technologies and techniques aim at improving the outcomes of ventral hernia repair, but recurrence remains a critical problem. Failure after hernia repair continues high, with recurrent reoperations at 12.3% after five years, and 23.1% at 13 years^1^.

LeBlanc and Booth first described laparoscopic ventral hernias (LVH) repair, in 1993. They demonstrated a feasible and safe intraperitoneal placement of expanded PTFE graft^2^. The LVH repair became attractive because of lower surgical wound morbidity compared to open techniques. Patients’ comorbidities and hernial morphology have been a problem, though. Long-term mesh complications are critical, and some times, their use is only to span the hernia defect without closing the fascia, denominated “bridged repair.” Meshes can generate infections, adhesions and intestinal fistulas, or even induce a severe problem when in need of abdominal reoperations. Furthermore, postoperative bulging in the operated area is usual, in orthostasis, or during physical activity^3^ ^-^ ^6^.

Open repairs to the abdominal wall, under certain conditions, seem more appropriate than minimally invasive procedures, regardless of using a mesh implant or not^7^ ^,^ ^8^. Another surgical proposal is the component separations (CS), an attractive hernia repair procedure. The surgeon carries out the incision of the abdominal wall with a musculoaponeurotic partitioning, reapproximating the fascia, and creating a dynamic abdominal wall. Despite providing an approximation of large defects, the CS can cause secondary weak areas, and disrupt the abdominal transverse muscle function. It requires additional dissection and it may damage the abdominal wall^9^.

Alcino Lazaro da Silva, in 1971, proposed the transposition with the hernial sac (THS) to ventral hernia repair, primarily in large midline, single elliptical defects, with an exuberant hernial sac^10^ ^,^ ^11^.

**Figure 1:**
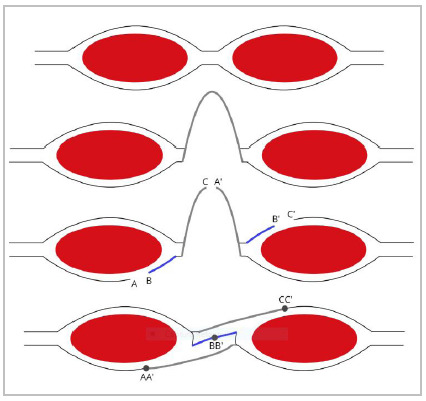
Schematic cross-sections of the abdominal wall, in the sequence from top to bottom: the normal aspect; showing hernia median incisional with the hernial sac; the bilateral and alternating relaxing incisions; and showing the transposition technique with the hernia sac (THS), by Lázaro da Silva. Lateral and medial leaflets posterior to the left (A and B) and the free edge of the left half of the sectioned hernia sac (C). The free edge of the right half of the bag (A’). Medial and lateral leaflets anterior to the right (B’and C’). Suture planes AA’, BB’ and CC’. Author: Guilherme Seronni, 2020.

THS is a set of relaxing, bilateral, and alternated incisions on the abdominal rectus sheath. The aponeurotic and hernial sac flaps are transposed, alternately, in three suture lines anchored in each other, retracing the alba line and the rectum sheath^12^ ^,^ ^13^ (Figure 1). Since Correa, in 1995, described a subcutaneous or pre-aponeurotic endoscopic approach and systematization, minimally invasive procedures have been feasible^14^ ^,^ ^15^. 

Recently Claus et al., in 2018, described the subcutaneous onlay laparoscopic approach (SCOLA) for ventral hernia and rectus abdominal diastasis repair^16^.

In this scientific communication, we describe the surgical steps of endoscopic hernial sac transposition, in a single patient. This minimally invasive procedure is a technical reproduction of a half-century open surgery which has been used for many year^10^. The study was approved by the local Research Ethics Committee.

## METHODS

### Case presentation

We report a healthy 48-year-old man undergoing an endoscopic peritoneal hernial sac transposition (eTHS) on a recurrent ventral hernia. He developed an incisional hernia after an abdominal trauma requiring a midline emergency laparotomy, three years before the eTHS. The patient underwent an open mesh ventral hernia repair 18 months after the abdominal trauma. Five months later, the hernia recurred after routine physical activity.

**Figure 2:**
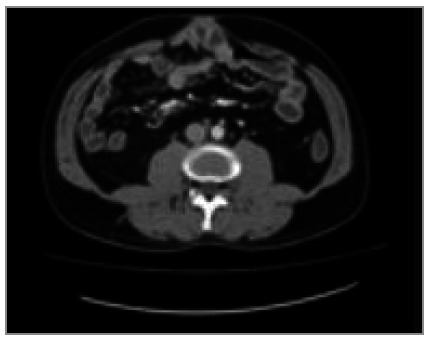
Preoperative abdominal tomography.

The patient’s physical examination confirmed a supraumbilical and midline adhered and exuberant scar tissue. The hernial defect was single and elliptical, reaching 9cm longitudinally and 5cm laterally (Figure 2). Preoperative abdominal tomography. After the clinical evaluation, the patient signed an informed consent form, agreeing with the operation, its risks and benefits, and the possibility of conversion to open surgery.

### Surgical technique

**Figure 3:**
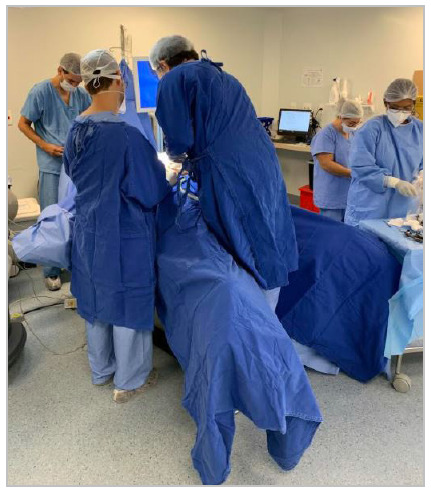
Position of the patient and the surgeon for the procedure.

The patient underwent general anesthesia, and two grams of intravenous cefazolin were administered at anesthetic induction. The patient was in the supine position with arms along the body, and the surgeon between the patient’s legs. A 30º Trendelenburg position was set, and then a hip extension defined a reverse V position, in order to minimize the surgeon’s hands touching the patient’s thighs, as well as allowing a wider space between the last rib and the iliac crest (Figure 3).

**Figure 4:**
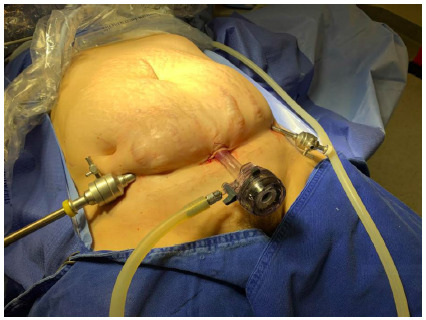
Position of the three suprapubic trocars.

We set three trocars in a line up along an imaginary path of the Pfannenstiel incision. The first one, a 20mm incision, transversal, and median, was carried out and reached the aponeurosis. Through a blunt dissection, we created an 8-10cm subcutaneous space laterally and towards the umbilicus. A 12mm trocar was inserted and fixed to the incision with a nylon suture avoiding CO_2_ ecaping. Then, two additional 5mm trocars were bilaterally positioned line up along the imaginary path (Figura 4).

The umbilicus was released from the abdominal wall and re-positioned at the end of the procedure. After the complete removal of the previous mesh which presented with a significant inflammatory process, we carefully preserved the entire hernia sac to used it for the hernia repair. After the entire ventral defect was exposed, we opened the hernial sac, which led to a pneumoperitoneum. Thus, an additional intraabdominal 5mm trocar was placed on the left flank.

We then repaired the defect in three layers. First, we performed an adhesiolysis on the left inner margin of the peritoneal hernial sac. A relaxing incision on the posterior left rectus sheath created two aponeurosis flaps, medial and lateral. The lateral rectus flap was a fixed by using a running suture with the right hernial sac flap.

After this, we performed a longitudinal incision on the right anterior rectus sheath, and the hernia defect was divided into two flaps, lateral and medial.

In the second layer, a running suture of the medial posterior rectus flap and medial anterior, rectus sheaths flap was carried out. This step is the most crucial reinforcement layer, as described by Melo^17^ ^,^ ^18^.

The last step of the transposition was between the right anterior rectus sheath lateral flap with the left flap of the hernial sac. We used 3-0 polydioxanone continuous suture, in all the three reinforcement layers. The pneumoperitoneum was deflated, and hemostasis carefully reviewed. A Blake’s drain was used for 72hours.

**Figure 5:**
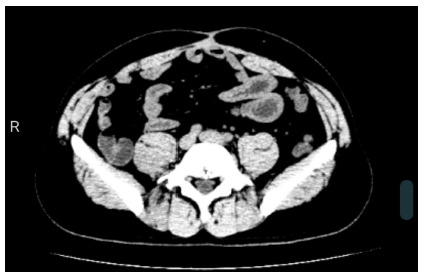
Postoperative abdominal tomography.

The patient was discharged on the first postoperative day, and was allowed to work. He returned for revision two weeks after surgery. The postoperative course was uneventful, and the patient was reassessed every six months without any symptoms. At 24 months follow-up, he remains healthy without any signs of recurrence. Interesting to note that he was involved in a motorcycle accident one year ago, presented with a broken right foot, and the eTHS repair remained intact, according to the clinical and tomographic exams (Figure 5).

## DISCUSSION

Incisional hernias are a common complication following abdominal operations, and represent about 80% of all ventral hernia^6^. Incisional hernia repair is a common surgical procedure and has long been a problem with no standard surgical treatment, and a significant postoperative morbidity. Laparoscopic repair has a lower postoperative morbidity. Nowadays, the use of laparoscopic ventral hernia repair is limited to patient and hernia factors^19^ ^,^ ^20^. 

The primary fascial repairs let to unacceptable recurrence rates as high as 46%^6^. Perhaps, the most revolutionary issue in incisional hernia surgery was tension-free fascial closure. However, it is often associated with synthetic mesh implants, with and without closing the defect^9^ ^,^ ^21^.

Mesh hernia repair became the gold standard for elective ventral hernia repair. Nonetheless, long-term complications of the intraperitoneal mesh and recurrence became a problem^5^ ^,^ ^22^ ^-^ ^24^. There is extreme underreporting and a lack of consistency of clinically relevant mesh properties. Thus, litigation issues after mesh implantation have increased all over the world^25^ ^,^ ^26^.

The tempting possibility of using the hernial sac as part of the incisional hernia repairs, associated with the relaxing of the incisions without damaging the abdominal wall was defended by Lázaro da Silva, more than 50 years ago^10^ ^-^ ^12^ ^,^ ^27^. Soft muscle fibers, vascular neoformation and heterotopic tissues were described as an autologous graft in the peritoneal hernial sac^28^ ^,^ ^30^. 

The starting point of this endoscopy approach was supported by the results of the open THS, over these 50 years. We have associated our personal experience with the open THS with the previous and extensive use of minimally invasive methods for most abdominal procedures. We believe that endoscopic access may be a significant factor for its popularity among laparoscopic surgeons. 

The clinical relevance of this study was that the fascial endoscopic approach provides a safe and anatomical repair of ventral hernia, with minimal morbidity.

Endoscopic access allowed for the safe and efficient treatment of the defect and the removal of the previously implanted mesh. In the present case, eTHS ventral hernia repair is a safe and reproducible technique.
